# Real-World Clinical Outcomes of Indigenous Biodegradable Polymer Drug-Eluting Stents

**DOI:** 10.7759/cureus.17886

**Published:** 2021-09-11

**Authors:** Basant Kumar, Raikot Rakesh Ram, Neelam Dahiya, Atit A Gawalkar

**Affiliations:** 1 Cardiology, Post Graduate Institute of Medical Education and Research, Chandigarh, IND; 2 Internal Medicine, Post Graduate Institute of Medical Education and Research, Chandigarh, IND

**Keywords:** bioabsorbable coronary stents, biodegradable coronary stents, indigenous stents, newer generation stents, coronary intervention

## Abstract

Introduction

The durable polymer has been shown to cause neoatherosclerosis, and chronic local inflammation, predisposing individuals to in-stent restenosis and stent thrombosis (ST). The biodegradable polymer stents, which degrade after the desired function of drug release is achieved, allow for endothelial healing. Indigenous coronary stent manufacturing and its use are on the rise nowadays, and their safety and efficacy have been studied in well-structured clinical trials. However, data are scarce on their safety and efficacy in the real-world clinical setting. In this study, we examine the real-world one-year performance of bioresorbable or polymer-free stents manufactured in India.

Materials and methods

This was a single-center, single-arm prospective observational study involving 210 patients undergoing intracoronary stenting using bioabsorbable or polymer-free drug-eluting stents (DES) from Indian manufacturers. All patients were followed up for 12 months prospectively for any major clinical events.

Results

The mean age of the enrolled patients was 57.04 years (IQR: 34-84 years), among which 159 (75.7%) were male; 99 (43.8%) patients had presented with acute myocardial infarction (MI). A total of 294 stents were deployed with a mean diameter of 3.1 ±0.4 mm, and a mean length of 29.4 ±9.1 mm. Two patients had experienced major adverse cardiovascular events (MACE). After three months of follow-up, one patient developed ST, and the same patient developed a cerebrovascular accident (CVA) after six months. After one year of follow-up, one patient died of cardiac causes.

Conclusion

Based on our findings, in the real-world clinical setting, the indigenously made biodegradable polymer DES are both safe and effective.

## Introduction

Intracoronary stent implantation emerged in 1980 for the emergency treatment of coronary dissection during angioplasty. However, it was plagued by high rates of subacute closure (3-5%) [1]. Subsequent improvements in the metal scaffold and stent design led to the popular bare-metal stents (BMS) outperforming isolated balloon angioplasty in all aspects of cardiovascular outcomes. But these bare-metal scaffolds still had high rates of restenosis and target vessel revascularization (TVR) to the tune of more than 10% in the first year [2,3]. The design of drug-eluting stents (DES), which was introduced in 2003, offered a solution to these high rates of restenosis. They had an antineoplastic drug mounted on a durable polymer [4]. These innovations led to thinner stent struts, newer antiproliferative drugs, and thinner polymer coats, which further improved cardiovascular outcomes [5,6]. However, neoatherosclerosis was seen with respect to these second-generation stents, which was primarily attributed to the residual polymer that delayed endothelial healing [7,8]. The persistent polymer stent coating has been shown to trigger a chronic inflammatory response, thereby delaying the process of stent coverage and hence predisposing the vessel to late thrombotic events. As the only function of the polymer is to act as a reservoir for the antiproliferative agent, stent designing with gradual resorption of the polymer began to reduce the inflammation, late stent thrombosis (ST), and neoatherosclerosis [9,10]. Hence, the bioabsorbable polymer stents were introduced, with SYNERGY (Boston Scientific, Marlborough, MA) being the first of its kind. After the controlled release of antiproliferative drug for a specified time, biodegradable polymer stents slowly degrade, allowing for endothelial healing. Subsequently, multiple bioabsorbable polymer stents have been seen to have similar TVR, ST, and major adverse cardiac events (MACE) as durable polymer scaffolds [11-13].

The last decade has seen significant growth in stent manufacturing in India. The price-capping of coronary stents imposed by the pricing authority has further stimulated the growth of indigenous stent manufacturing, and the market shares of imported stents have consequently shrunk as per anecdotal reports [14]. Various Indian manufacturers produce biodegradable or polymer-free stents that are widely used across the world. The safety and efficacy of these Indian stents have been established in well-structured clinical trials [15-22]. However, there is a scarcity of data and evidence related to their safety and efficacy in the real-world or clinical setting. In this study, we analyze the real-world one-year performance of various bioresorbable or polymer-free stents manufactured in India.

## Materials and methods

This was a single-center, single-arm prospective observational study carried out at a tertiary care center. Patients who underwent percutaneous coronary angioplasty between July 2019 and December 2020 using bioabsorbable or polymer-free drug-eluting coronary stents from Indian manufacturers were included. The study was reviewed and cleared by the ethical committee (Institutional Ethics Committee, Post Graduate Institute of Medical Education and Research, Chandigarh; IRB no. INT/IEC/2021/SPL-185). Patients above 18 years of age who underwent coronary stenting for any indication as per the society guidelines were included. Subjects who refused to provide consent and those who died during index hospitalization for reasons not related to coronary stent were excluded. Informed consent was taken from each subject.

Significant clinical events that occurred one year after index percutaneous coronary stenting were recorded prospectively during follow-up hospital visits or telephonically. Our study did not influence any clinical decisions, and no additional investigations or procedures were done as a part of the study. The operator had the discretion to decide the specific indigenous stent that was deployed for a particular patient based on the requirement of stent size, flexibility, and trackability. The length and diameter of the stent and the angioplasty technique were based on the operator's discretion, depending on the lesion characteristics. The stents used were Vivo Isar (sirolimus and probucol-eluting polymer-free DES; Translumina Therapeutics, New Delhi, India), Tetrilimus (biodegradable polymer, everolimus-eluting DES; Sahajanand Medical Technologies Ltd, Ernakulam, India), Tetriflex (biodegradable polymer, sirolimus‐eluting DES; Sahajanand Medical Technologies Ltd), Yukon Choice Flex (biodegradable polymer, sirolimus‐eluting DES; Translumina Therapeutics), Evermine (ultrathin platform, biodegradable polymer, everolimus-eluting DES; Meril, Vapi, India), BioMime (biodegradable polymer, sirolimus‐eluting DES; Meril), Abluminus (biodegradable polymer, sirolimus-eluting DES; Concept Medical India, Surat, Gujarat), and Pronova XR (polymer-free, sirolimus‐eluting DES; Vascular Concepts Limited, New Delhi, India). All patients were followed up prospectively after the index procedure in the outpatient clinic or telephonically (given the COVID-19 pandemic during the study period). All the patient data were entered into Microsoft Excel, and statistical analyses were done using SPSS Statistics version 26 (IBM, Armonk, NY). Categorical data were presented as proportions, and continuous data were presented as mean ±SD (parametric) and median (IQR) (non-parametric).

## Results

A total of 210 patients were enrolled in the study. The median age was 57.04 years (IQR: 34-84 years), among which 159 (75.7%) were male. Five patients (2.4%) were above the age of 75 years. Ninety-two (43.8%) were diabetic, 130 (61.9%) were hypertensive, and 91 (43%) were smokers. Ninety-nine (43.8%) patients presented with acute myocardial infarction (MI), 35 (16.7%) patients presented with unstable angina, and rest with stable ischemic heart disease. Thirty-two (14.7%) subjects were patients of young MI (males aged below 50 years and females aged below 60 years) (Table 1).

**Table 1:** Baseline demographic and clinical characteristics of the study population IQR: interquartile range; MI: myocardial infarction

Baseline characteristics	Values
Age in years, mean (IQR)	57 (34-84)
Age-wise distribution, n (%)	
<50 years	46 (21.1%)
50-75 years	159 (75.8%)
>75 years	5 (2.4%)
Sex, n (%)	
Male	159 (75.7%)
Female	51 (24.3%)
Smoking (active or former), n (%)	134 (65.2%)
Diabetes, n (%)	92 (43.8%)
Hypertension, n (%)	130 (61.90%)
Indication, n (%)	
Stable angina	76 (36.1%)
Unstable angina	35 (16.6%)
Acute MI	99 (47.1%)
Young MI	32 (15.2%)

Forty-nine (23.3%) patients had triple vessel disease (TVD). Out of 223 lesions involved, 116 (57.2%) were in the left anterior descending artery (LAD), 41 (20.2%) were in the left circumflex artery (LCX), and 66 (32.5%) were in the right coronary artery (RCA). A total of 294 stents were deployed, with a mean stent-per-patient rate of 1.38 ±0.59. A single stent was deployed in 146 patients, two in 56, and three stents in 12 patients. The mean diameter of the stents deployed was 3.1 ±0.4 mm, and the mean length of the stent deployed per lesion was 29.4 ±9.1 mm (Table 2).

**Table 2:** Angiography and angioplasty characteristics of the study population PCI: percutaneous coronary intervention; LAD: left anterior descending artery; LCX: left circumflex artery; RCA: right coronary artery

Angiographic and intervention characteristics	Values
Single vessel disease, n (%)	95 (45.2%)
Triple vessel disease, n (%)	49 (23.3%)
PCI vessel, n (%)	
LAD	116 (57.2%)
LCX	41 (20.22%)
RCA	66 (32.55%)
Number of stents deployed per patient, n	
1	141
2	56
3	12
Number of stents per patient (mean +SD)	1.4 ±0.6
Coronary stents (mean +SD)	
Stent diameter	3.1 ±0.4 mm
Stent length	29.4 ±9.1 mm
Stents used, n (%)	
Vivo Isar	29 (14.5%)
Tetrilimus	89 (44.7%)
Tetriflex	8 (4%)
Yukon Choice Flex	40 (20%)
Evermine	24 (12%)
BioMime	1 (0.5%)
Abluminus	6 (3%)
Pronova XR-8	2 (1%)

All the patients were followed up for 12 months prospectively. A total of three follow-up interviews were conducted at three, six, and 12 months. Two MACEs (defined as any ST, TVR, and death) were recorded during the follow-up period, including one ST presenting as MI and one death. After three months of follow-up, one patient developed ST, the same patient developed CVA after six months of follow-up. One patient died of cardiac cause at one-year follow-up (Table 3). MI due to ST occurred in a 43-year-old male diabetic, who underwent stenting of LAD when he first presented with non-ST elevation MI (NSTEMI). Two stents (Tetriflex, 2.5 x 20 and 3 x 34 mm) were deployed in LAD. He presented to us with ST at the mid-segment of LAD at three months. We revascularized the patient. He subsequently developed a stroke at six months despite undergoing dual antiplatelet therapy. Sudden cardiac death occurred in a 50-year-old male non-diabetic, who underwent stenting of LAD using Yukon Flex Choice (3.5 x 40 mm) for anterior wall MI. He did not have any lesions in the other coronary arteries.

**Table 3:** Clinical outcomes of study subjects on follow-up CV: cardiovascular; MI: myocardial infarction: MACE: major adverse cardiovascular events

Outcome	Values
Primary outcome (composite of MACEs)	2 (0.92%) events
Total CV death	1 (0.46%)
Total stent thrombosis-MI	1 (0.46%)
Events at 3-month follow-up	
Stent thrombosis	1 (0.46%)
Events at 12-month follow-up	
Death	1 (0.46%)

## Discussion

Many studies have shown that residual polymer after drug elution can cause an inflammatory response and delay endothelial healing, thereby leading to increased incidence of late ST and in-stent restenosis [9,23,24]. Biodegradable polymer stents provide the solution to this by not only effectively delivering the drugs while in the coronary artery but also by completely self-degrading into harmless compounds such as water and carbon dioxide after drug release has been completed [25]. Recent decades have seen dramatic growth in stent innovation and the manufacturing of indigenous coronary stents with biodegradable polymers. Various studies have shown the feasibility and efficacy of these stents individually [16-22,26]. However, there is a scarcity of literature regarding the real-life experience related to these stents outside of trial settings, especially outcome analysis with these stents examined together. Our study highlights the safety and efficacy of these indigenous stents in the clinical setting and their comparison with the findings of similar studies.

The most common stents implanted were Tetrilimus (30%) and Yukon Choice Flex (13.6%); hence we compared the results of our findings with those of similar studies conducted with these stents. Similar to our work, the See-Real Registry studied the real-world one-year outcomes in patients stented with Tetrilimus (ultra-thin, biodegradable polymer, everolimus-eluting stent), and Xhepa et al. reported on the one-year real-world clinical outcomes in patients who underwent coronary angioplasty with Yukon Choice Flex (biodegradable polymer, sirolimus-eluting stent) [17,27]. The mean age group of our study population was 57 ±9.9 years, whereas the mean age in the See-Real Registry was 57.5 ±11.9 years while that in the Yukon Choice Flex study was 68.2 ±10.7 years. In our study, patients with diabetes constituted 43.8% (n=92) of the cohort, which is higher than those in the studies mentioned above (around 25%). The most common indication of stenting in our population was acute MI (47.1%), which is higher than the Yukon Choice Flex study (25.4%). The patients presenting with TVD in our study population was 23.3%, which was lower when compared to other studies. The mean length of the stent deployed was 29.4 ±9.1 mm while the mean diameter was 3.1 ±0.4 mm, which are in line with the compared studies.

ST leading to MI was seen in only one subject (0.46%), whereas two subjects (0.3%) developed ST in the Yukon Choice Flex study. The same patient underwent TVR. The incidence of ST was 0.7% in the Supraflex arm of the TALENT study, where the biodegradable polymer Supraflex stent was compared with durable polymer Xience stent. One patient in our study died (0.46%), and given the sudden nature of the death, it was likely due to a cardiac cause. Death was noted in 2.4% (n=19) of patients in the Yukon Choice Flex study, in 1% (n=7) in the Supraflex arm, and 0.3% (n=2) in the Xience arm of the TALENT study. The total number of MACEs in our study was two (0.92%), which is much lower compared to the See-Real Registry, which observed MACEs in 4.3% (n=9) of its subjects. The incidence of MACEs in the Supraflex arm of the TALENT study was 4.9% (n=35), and it was 5.3% (n=37) in the Xience arm of the TALENT study.

The number of significant events in our study was relatively lower than those in similar studies in real-life and trial scenarios. This could be attributed to various factors such as a smaller study population, shorter duration, lower number of TVD patients, and a higher proportion of young MI. Our study is limited by the small number of study subjects, short-term follow-up, and the fact that it was conducted at a single center; moreover, the study was observational in nature. The total number of events was too small to make any significant assumptions and recommendations.

## Conclusions

The outcomes in our study have been compared with the previous reports in terms of the efficacy of the stents. The newer bioabsorbable or biodegradable stents have good short-term clinical outcomes and can overcome the shortcomings of persistent polymers. The demonstration of efficacy in a clinical setting where stents from multiple manufacturers are used further reinforces their efficacy in the real-world setting. The study has shown that in the real-world scenario, the indigenous biodegradable polymer DES are both safe and effective. Further prospective randomized studies involving similar populations and stent types are warranted to validate our findings on the safety and efficacy.
